# Neutrophil extracellular traps promote thrombogenicity in cerebral venous sinus thrombosis

**DOI:** 10.1186/s13578-022-00845-z

**Published:** 2022-07-22

**Authors:** Jiaqi Jin, Shan Qiao, Jie Liu, Wenqiang Li, Fang Wang, Xin Gao, Jiawei Tian, Nan Wang, Jiheng Zhang, Jiawei Dong, Haiyun li, Jianjun Wang, Shaoshan Hu, Peng Zhou

**Affiliations:** 1 Department of Neurosurgery, The Second Affiliated Hospital of Harbin Medical University, Harbin, China; 2 Department of Neurosurgery, Emergency Medicine Center, Zhejiang Provincial People’s Hospital, Hangzhou Medical College, Hangzhou, Zhejiang China; 3 Department of Neurology, The First Affiliated Hospital of Shandong First Medical University & Shandong Provincial Qianfoshan Hospital, Jinan, China; 4 Department of Vascular Surgery, Jinshan Hospital of Fudan University, Shanghai, China; 5 Department of Neurology, Qilu Hospital, Cheeloo College of Medicine, Shandong University, Jinan, China; 6 Department of Neurosurgery, The First Affiliated Hospital of Shandong First Medical University & Shandong Provincial Qianfoshan Hospital, Jinan, China

**Keywords:** Cerebral venous sinus thrombosis, Neutrophil extracellular traps, Platelet factor 4, Coagulation, Endothelial dysfunction

## Abstract

**Background:**

Neutrophil extracellular traps (NETs) contribute to the creation of a coagulation state in various diseases. Currently, it is not clear whether NETs are present in the thrombi and plasma of patients with cerebral venous sinus thrombosis (CVST). This study aimed to investigate the presence of NETs in thrombi and blood samples from CVST patients and the procoagulant activity (PCA) of NETs during the progression of CVST.

**Results:**

Thrombi obtained from CVST patients undergoing thrombectomy were examined by immunochemistry using neutrophil elastase (NE), CD66b and citrullinated histone H3(citH3). The presence of NET markers in samples from 37 CVST patients and 32 healthy people was evaluated by ELISA. NET-producing neutrophils and neutrophil-platelet (PLT) aggregates were examined in samples obtained from CVST patients and healthy people by flow cytometry. The TAT complex in plasma sample from each group was detected by ELISA to evaluate the procoagulant activity of NETs in CVST patients. Neutrophils from healthy subjects were treated with PLT-rich plasma in the presence of anti-PF4 antibodies or an autophagy inhibitor and analyzed by flow cytometry and confocal microscopy. After treatment with NETs, the expression of von Willebrand factor (VWF), tissue factor (TF) and CD31 in human brain microvascular endothelial cells (HBMECs) was measured by confocal microscopy and western blotting. Our results showed that NETs were abundant in the plasma and thrombi from CVST patients. Platelet factor 4 (PF4) from CVST PLTs induced NET generation through autophagy. NETs could induce PCA by modulating TF and phosphatidylserine (PS) in CVST. NETs also disrupted the endothelial barrier and transformed ECs into a procoagulant phenotype to exacerbate thrombogenicity.

**Conclusions:**

NET generation was mediated by PF4 from PLTs through autophagy and contribute to thrombosis in CVST patients.

**Supplementary Information:**

The online version contains supplementary material available at 10.1186/s13578-022-00845-z.

## Introduction

Cerebral venous sinus thrombosis (CVST) is a rare, life-threatening cerebrovascular disease with an estimated annual incidence of 3-to-4 cases per 2 million adults [1–3]. Several risk factors for CVST have been identified, including brain tumors, cancer, cerebral infections or traumas, oral contraceptive use, pregnancy, the postpartum period, and thrombophilia [2, 3]. However, the predisposing factors for CVST in 15–20% of patients have not yet been elucidated. Due to a wide spectrum of clinical presentations, the misdiagnosis rate of CVST is relatively high. Moreover, CVST carries an approximate mortality rate of 6–10%, and the mortality rate of severe CVST is as high as 30% [4, 5]. Therefore, it is urgent to elucidate the underlying mechanism of CVST.

Neutrophils are the most abundant immune cells in the circulation and play a critical role in disease pathogenesis, inflammation, and tissue injury [6]. Moreover, neutrophils shift the coagulation balance in favor of thrombus formation through the generation of extracellular chromatin structures called neutrophil extracellular traps (NETs) [7, 8]. NETs are composed of extracellular strands of decondensed DNA complexed with histones and neutrophil granule proteins such as myeloperoxidase (MPO) and neutrophil elastase (NE) [9, 10]. NETs have been shown to play a pivotal role in thrombus formation and are a potential therapeutic target for thrombolysis [11–13]. However, evidence of the presence and the definite role of NETs in CVST is scarce.

A recent study reported the presence of NETs in CVST patients who received the coronavirus (COVID-19) vaccine [14, 15]. However, the sample size in this study was relatively small, and the CVST patients enrolled were only those who received the COVID-19 vaccine, which may limit the conclusions drawn. Moreover, the specific mechanism of NET generation in CVST is poorly understood. Platelet factor 4 (PF4) is stored in platelet (PLT) granules and was identified as an important player in thrombotic diseases in previous studies [16, 17]. PF4 in activated PLTs has also been shown to induce NET formation in some diseases [18]. However, relatively little is known about the interaction between PF4 and NET formation in CVST.

Herein, we explored the presence of NETs in thrombi and plasma samples from CVST patients. Moreover, the potential role of PLTs in NET formation was evaluated. As endothelial activation is critical in thrombus formation [19–21] and NETs have been studied to exert cytotoxic effects on endothelial cells (ECs) in previous reports [22–24], we further explore the association between NETs and HBMECs in CVST. Our study may help to identify the potential mechanisms of thrombosis in CVST and identify novel therapeutic targets for intervention and prevention in CVST patients.

## Materials and methods

### Patients

We selected 32 healthy individuals and 37 CVST patients who were admitted to the First Affiliated Hospital of Shandong First Medical University and Qilu Hospital of Cheeloo College of Medicine, Shandong University, from October 2018 to May 2021. CVST was diagnosed based on definite diagnostic criteria and computed tomography (CT) or magnetic resonance imaging (MRI) with contrast-enhanced venography. Patients who had experienced symptoms within 12 h prior to admission were eligible for inclusion and received thrombectomy. Exclusion criteria included patients with hematological disease, infections, liver or renal failure and malignancy. The main characteristics of the patients and healthy controls are shown in Table 1. This study was approved by the Ethics Committee of The First Affiliated Hospital of Shandong First Medical University and Qilu Hospital of Cheeloo College of Medicine and was conducted according to the Declaration of Helsinki.

**Table 1:** The main clinical and laboratory features of 32 healthy subjects and 37 patients diagnosed with CVST

Characteristics	Control (n = 32)	CVST (n = 37)
Age (years)	35.15 ± 11.4	34.27 ± 9.49
Male (n, %)	22(65.6%)	25(67.6%)
WBC (× 10^9^)	6.15 ± 1.40	7.38 ± 2.94*
Neutrophils (%)	58.08 ± 10.75	70.14 ± 10.53*
Monocytes (%)	35.63 ± 9.59	21.10 ± 9.36*
Lymphocytes (%)	4.35 ± 1.71	6.93 ± 2.64*
Esoinophils (%)	1.84 ± 1.14	1.34 ± 1.60
Basophils (%)	0	0.46 ± 0.97
Erythrocytes(× 10^12^/L)	4.62 ± 0.41	4.30 ± 0.87
Hg (g/L)	136.55 ± 15.24	124.43 ± 26.92*
PLT (× 10^9^)	237.37 ± 62.24	224.87 ± 97.80*
PT (s)	10.99 ± 0.40	13.97 ± 4.70*
APTT (s)	34.75 ± 3.45	35.94 ± 17.88
D-dimer (g/L)	0.05 ± 0.03	1.55 ± 4.41*
Fibrinogen (mg/L)	2.61 ± 0.75	3.97 ± 2.24*
Thrombosed sinus and veins		
Superior sagittal sinus	–	12 (32.4)
Transverse sinus	–	6 (16.2)
Sigmoid sinus	–	6 (16.2)
Straight sinus	–	4 (10.8)
Deep venous system	–	4 (10.8)
Jugular vein	–	4 (10.8)
Perficial venous system	–	12 (32.4)
2 sinuses involved	–	10 (27.0)
Treatments		
Surgery	–	17(45.9)
Non-surgery	–	20(54.1)

*WBC* white blood cells, *Hb* hemoglobin, *PLTs* platelets. Data are presented as numbers (percentages) or the median ± SD. *P < 0.05 vs. healthy control

### Human samples

Plasma was obtained from healthy volunteers and patients with a clinical diagnosis of CVST. Two kinds of venous blood samples were obtained from each CVST patient: one was a peripheral venous sample, and the other was from the culprit venous sinus during stent retriever thrombectomy (venous sinus thrombus site sample). A 15 mL whole blood sample from the area surrounding the venous sinus thrombus was obtained through the thrombotic material. The venous sinus thrombus was immediately transferred to 4% paraformaldehyde (PFA), paraffinized within 24 h, embedded in OCT, and stored at −80 °C until use. PLT-rich plasma (PRP) was obtained via centrifugation at room temperature (150 × g for 15 min) and was immediately used for co-incuabtion experiments after being isolated. Neutrophils were isolated using human neutrophil separation medium (TBD, Tianjin) according to the manufacturer’s instructions [17]. The isolation of PLTs was performed as previously described [25].

### NET assays

The isolation of NETs was performed as previously described [26]. Quantification of MPO-DNA, NE-DNA, citrullinated histone H3 (citH3)-DNA, phosphatidylserine (PS)-DNA and tissue factor (TF)-DNA was performed using modified enzyme-linked immunosorbent assay (ELISA) kits [22]. Fifty microliters of plasma was added to 96-well microtiter plates coated with MPO (human MPO ELISA kit, Jingkangbio, Shanghai), NE (human NE ELISA kit, Jingkangbio, Shanghai), citH3 (human, citH3 ELISA kit, Jingkangbio, Shanghai), PS (human PS ELISA kit, Jianglaibio, Shanghai), and TF (human TF ELISA kit, Cloud clone, Houston) as the capture antibody and incubated at 37 °C for 1 h. After being blocked in 1% bovine serum albumin (BSA), a Quant-iT PicoGreen dsDNA assay kit (Invitrogen) was used to examine each well according to the manufacturer's instructions. The final DNA concentrations were defined as NET-DNA concentrations.

### PLT‑neutrophil coculture assay

To investigate the interaction between activated PLTs and NETs, a PLT-neutrophil coculture system was established [18]. Neutrophils were seeded on 24-well plates at 1 × 10^5^ cells per well and incubated with PRP or PLTs (2 × 10^6^ cells per well) from each group for 1 h. NET formation was quantified by ELISA (citH3-DNA). Recombinant PF4 protein (Elabscience), anti-PF4 antibodies (Affinity, 5 μg/mL) and HCQ (Suolaibio, 50 μM) were used to evaluate whether PF4 induced NET formation through autophagy.

### EC stimulation assays

The human brain microvascular endothelial cell (HBMEC) line (hcMEC/D3) was purchased from Yuchunbio (Shanghai) and authenticated Shanghai by Biowing Applied Biotechnology Co. Ltd. via STR profiling. The STR profiles match the standards recommended for cell line authentication. HBMECs were incubated with neutrophil-conditioned medium from healthy individuals and CVST patients in the presence of DNase I (100 U/mL, Thermo Fisher) for 4 h. To detect the cytotoxicity of NETs on HBMECs, HBMECs (1 × 10^5^ cells/ml) were incubated for 4 h with isolated NETs (0.1 μg DNA/mL or 0.5 μg DNA/mL) in the presence of DNase I (100 U/mL, Thermo Fisher), activated protein C (APC, 100 nM, Med Chem Express), and sivelestat (100 nM, Med Chem Express). Fibrin formation by ECs was examined as previously described [24].

### Flow cytometry

Approximately 100 μL of citrated blood was freshly collected from healthy subjects and CVST patients, diluted in PBS (1:1) and stained with anti-CD15 (PE, Biolegend, 301906), anti-CD66b (APC-Cy7, Bioledgend, 305125), anti-CD41 (PerCP-Cy5.5, Biolegend, 303720), anti-citH3 (ab5103, 1 μg/mL), and anti-MPO (Proteintech, 1 μg/mL), followed by incubation with goat anti-rabbit secondary antibodies conjugated to Alexa Fluor 488 (ab150081, 1 μg/mL) and goat anti-mouse secondary antibodies conjugated Alexa Fluor 647 (ab150115, 1 μg/mL). The treated PLTs were incubated with anti-CD62P antibodies (APC, BD304910). Flow cytometry was used to examine NET-producing neutrophils and PLT-neutrophil aggregates (NPAs) as previously described [27]and showed in supplemental Fig. 1. To measure the expression of TF on ECs, treated ECs were stained with anti-CD142 antibodies (APC, Biolegend 365,205). Samples were fixed with 1% PFA and analyzed by flow cytometry (BD, FACSCanto™ II).

**Fig. 1:**
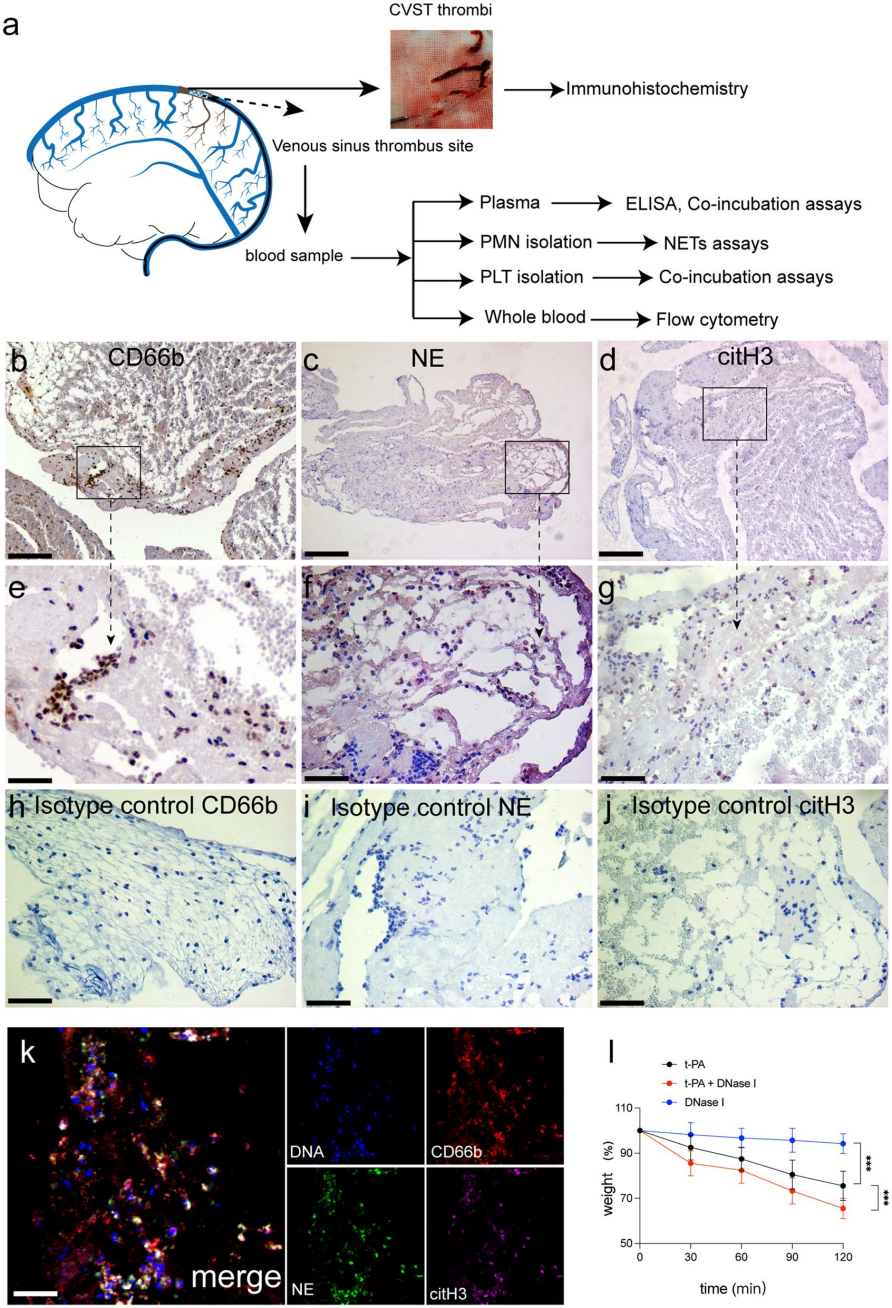
NETs are abundant in CVST thrombi. **a** A schematic drawing of thrombi and blood samples during stent retriever thrombectomy. **b**–**g** Thrombi sections obtained from CVST patients were stained with antibodies against CD66b, NE and citH3. Immunohistochemical staining showed that the granulocytes in thrombi were neutrophils stained with NE, and the presence of NETs in CVST thrombi was confirmed by the high expression of citH3. **h**–**j** Isotype control staining of CD66b, NE, and citH3 signal in thrombi samples from CVST patients. **k** The Thrombi sections were stained with CD66b (red), citH3 (pink), NE (green) and DAPI (blue) and analyzed by confocal microscopy. **l** Two equal parts of fresh patient thrombi were subjected to ex vivo lysis by tPA (1 mg/ml), DNase I (100 U/ml), or a combination of the two for 120 min. The combination of DNase I and tPA markedly promoted ex vivo lysis compared to tPA or DNase I alone, reducing the thrombi weight up to 34.5% at 120 min. Statistics: Repeated measures analysis of variance. The inset scale bars in b-d and h–k are 20 μm and e–g are 40 μm. ***P < 0.001

### Immunofluorescence imaging

To conform the presence of NETs, neutrophils were incubated with DAPI (4,6-diamidino-2-phenylindole), anti-histone H3 (citrulline R2 + R8 + R17, Novus, 1 μg/mL), and anti-MPO antibodies (ab25989, 1 μg/mL). To colocalize NETs with PS and TF, treated neutrophils were stained with anti-TF antibodies (ab48647, 1 μg/mL) and FITC-conjugated bovine lactadherin (Hematologic Technologies, 1 μg/mL). ECs were cultured on fibronectin-coated slide flasks and stained with anti-CD31 (ab9498, 1 μg/mL), anti-von Willebrand factor (vWF, Proteintech, 1 μg/mL), anti-ICAM-1 (Proteintech, 1 μg/mL), anti-VCAM-1 (Proteintech, 1 μg/mL), and anti-TF antibodies (ab48647, 1 μg/mL). The frozen tissue samples were cut into 8 μm sections and stained with anti-histone H3 (citrulline R2 + R8 + R17, ab5103, 1 μg/mL), anti-CD66b (Proteintech, 1 μg/mL), anti-Beclin-1(Proteintech, 1 μg/mL), anti-PF4(Proteintech, 1 μg/mL) and anti-LC3B (Proteintech, 1 μg/mL) antibodies. For co-localization of citH3 with CD66b and PF4 or autophagy markers in thrombi sections, we obtained Alexa Fluor 647 Conjugated citH3 antibody performed with Alexa Fluor 647 Conjugation Kit (Fast)—Lightning-Link (ab269823) and anti-histone H3 (citrulline R8, ab232939), according to instructions. The sections were analyzed by confocal microscopy (Zeiss, LSM 800).

### Ex vivo lysis of thrombi

Thrombi were obtained from CVST patients and placed in PBS, and 2 equal parts of the fresh thrombi were subjected to ex vivo lysis experiments as previously described [13].

### Immunohistochemistry

Frozen sections of thrombi from CVST patients were cut into 8 μm sections and washed in PBS for 20 min. Then the thrombus sections were blocked with 10% goat serum and 1% BSA for 1 h, followed by overnight incubation at 4 °C with anti-histone H3 citrulline (R2 + R8 + R17, ab5103, 1 μg/ml), anti-CD66b (Affinity, 1 μg/mL) and anti-NE antibodies (ab131260, 1 μg/ml). After endogenous peroxidase activity was blocked, the sections were incubated with horseradish peroxidase (HRP)-conjugated secondary antibodies according to the manufacturer's protocol.

### ELISA

PF4, thrombin-antithrombin (TAT) complex and von Willebrand factor (VWF) in plasma samples were detected by TAT complex ELISA kit (Elabscience, Shanghai), PF4 ELISA kit (Elabscience, Shanghai) and vWF ELISA kit (Elabscience, Shanghai), respectively. The TAT complex inhibition assay was performed as previously described [22]. After being incubated with endonuclease, MPO, NE, and citH3 levels in tissue supernatant were measured by an MPO ELISA kit (Jingkang, Shanghai), NE ELISA kit (Jingkang, Shanghai), and citH3 ELISA kit (Jingkang, Shanghai), respectively.

### Statistical analysis

Shapiro–Wilk test was used to test normal distribution of the datasets. To compare two groups, Wilcoxon test and paired t test were used for paired samples and Mann–Whitney and Unpaired t test with Welch’correction were used for unpaired samples as needed. For comparisons among more groups, ordinary one-way ANOVA, Brown-Forsythe and Welch’s ANOVA test and Kruskal–Wallis test and repeated measures analysis of variance were used as needed. All analyses were performed with Prism 9.0. A P value < 0.05 was considered statistically significant.

## Results

### NETs are abundant in CVST thrombi

The overall workflow for blood samples and thrombus tissue specimens from CVST patients who underwent endovascular thrombectomy processing is shown in Fig. 1a. To confirm the presence of NETs in CVST thrombi, thrombus sections were stained with CD66b (neutrophil marker), NE (neutrophil marker), and citH3 (NET marker). Immunohistochemical analysis showed obvious expression of CD66b, NE, and citH3 in thrombi sections, indicating the presence of NETs (Fig. 1b–j). Immunofluorescence staining also revealed that NETs colocalized with CD66b and citH3 in thrombi and covered a major portion of granulocytes (Fig. 1k). After confirming the presence of NETs in thrombi, it was also necessary to determine whether the pharmacological breakdown of NETs could enhance thrombi dissolution. Thus, ex vivo lysis of two equal parts of fresh patient thrombi was performed using tPA (1 mg/mL), DNase I (100 U/mL), or a combination of the two for 120 min. We found that the combination of DNase I and tPA markedly promoted ex vivo lysis compared to tPA or DNase I alone, reducing thrombi weight up to 34.5% at 120 min (Fig. 1l).

### Neutrophils are primed to form NETs in CVST patients

**Fig. 2:**
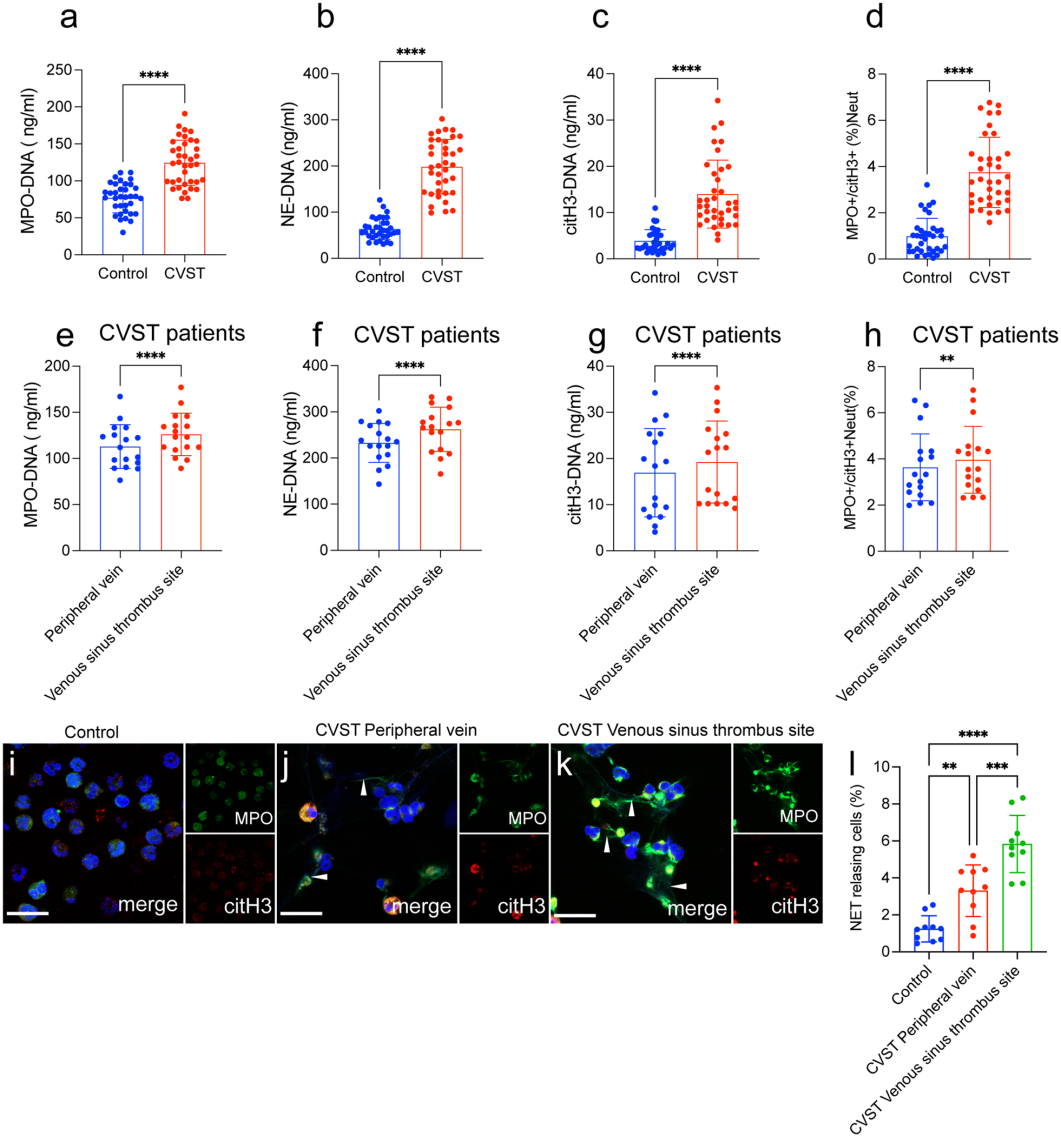
Neutrophils form extracellular traps in CVST patients. **a**–**c** MPO-DNA, NE-DNA, and citH3-DNA were measured in peripheral blood plasma from healthy subjects (n = 35) and CVST patients (n = 37). NET markers in peripheral venous blood samples from CVST patients were significantly higher than those from healthy subjects. **d** Flow cytometry was used to examine NET-producing neutrophils (MPO^+^/citH3^+^Neut) in samples from healthy controls (n = 32) and CVST patients (n = 37). **e**–**g** NET markers were measured in peripheral blood plasma (n = 17) and the venous sinus thrombus site from the same patients (n = 17). **h** The rate of NET-releasing cells from CVST peripheral blood and CVST venous sinus thrombus site from the same patients(n = 17). **i**–**l** Neutrophils isolated from healthy people (n = 10) and CVST patients (peripheral blood plasma and the venous sinus thrombus site, n = 10) were stained with MPO (green) and citH3 (red) and observed by confocal microscopy. Statistics: Unpaired t test with Welch ‘correction (**a**); Mann–Whitney (**b**, **d**); Wilcoxon t test **c**, **g**, **h**; paired t test (**a**, **e**, **f**); Ordinary one-way ANOVA **(i)**. The inset scale bars in E–G are 40 μm. **P < 0.01 and ****P < 0.0001. Neut, Neutrophils

Based on above findings, we further investigated the NETs formation in circulating blood in CVST patients. The NET markers MPO-DNA, NE-DNA, and citH3-DNA were measured in the plasma samples from healthy people and CVST patients (Fig. 2a–c). The ELISA results showed that these NET markers showed an obvious elevation in peripheral blood samples from CVST patients (n = 37) compared with those from healthy subjects (n = 32). In addition, NET-producing neutrophils, which were defined as MPO^+^/citH3^+^neutrophils (Neut), in blood samples from healthy people and CVST patients and analyzed by flow cytometry (Fig. 2d). The level of NET-producing neutrophils was the highest among these groups, further confirming NET generation in CVST patients (Fig. 2d). Thrombectomy was used to obtain blood samples from the venous sinus thrombus site. and NET markers were compared between venous sinus thrombi and peripheral venous blood from the same patients (n = 17). Interestingly, we observed that NET markers and MPO^+^/citH3^+^ cells were higher in samples from the venous sinus thrombus than in those from peripheral venous blood (Fig. 2e–h). Moreover, neutrophils from each group were stained with MPO (neutrophil marker) and citH3(NET marker) to confirm the presence of NETs and were observed by confocal microscopy (Fig. 2i–l). Confocal images showed that neutrophils in samples from the venous sinus thrombus site tended to generate higher levels of NETs than those from the other groups (Fig. 2i–l). Our findings revealed that the generation of NETs in circulation was increased in CVST patients and local venous sinus thrombus site seemed to more tend to trigger NETs formation, suggesting the thrombotic role of NETs in CVST patients.

### PF4 from activated PLTs induces NET formation through autophagy

**Fig. 3:**
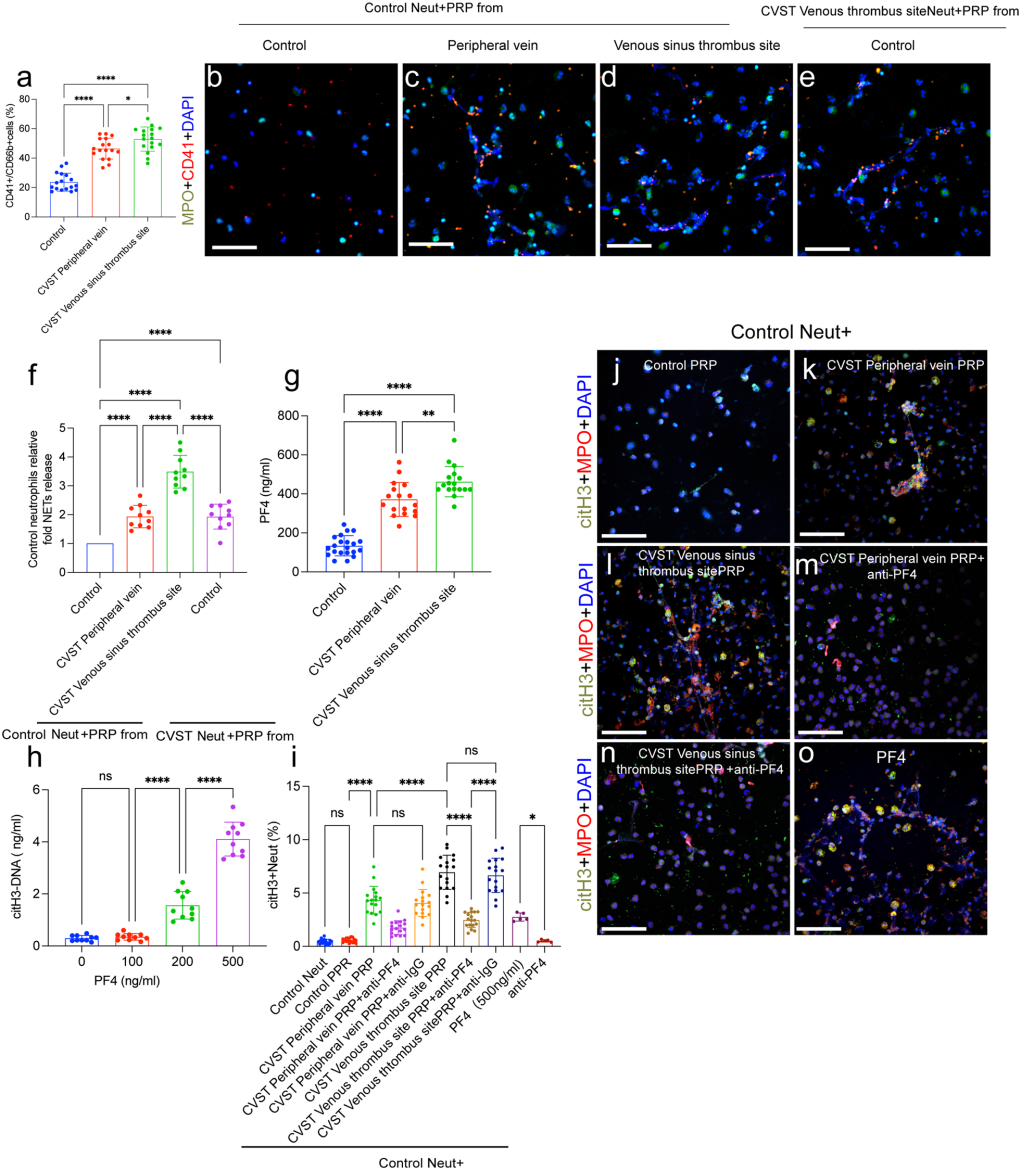
PF4 induces NET formation in CVST patients. **a** NPAs (CD41^+^/CD66b^+^) were examined in samples from healthy controls (n = 20) and CVST (n = 17) patients. **b**–**e** Neutrophils (from healthy people) were incubated with PRP from patients and healthy subjects and stained with CD41 (red) and MPO (green) to show the colocalization of NETs and PLTs. **f** Neutrophils (from CVST patients) were incubated with PRP from healthy subjects and stained with CD41 (red) and MPO (green). **g** Plasma PF4 levels were measured in samples from CVST patients (n = 37) and healthy subjects (n = 32). **h** Control neutrophils were treated with different concentrations of recombinant PF4 protein. CitH3-DNA was measured in each group (n = 10). Control neutrophils were incubated with PRP from healthy subjects and CVST patients in the presence of anti-PF4 antibodies. **i** NET releasing neutrophils (citH3 + Neut) was measured in cells from each group by flow cytometry. **j**–**o** Neutrophils were stained with MPO (red) and citH3 (green). Statistics: Ordinary one-way ANOVA (a, g); Brown-Forsythe and Welch’s ANOVA test (**f**, **h**, **i**). The inset scale bars are 20 μm in **b**–**e**, **j**–**o**. *P < 0.05, **P < 0.01, ***P < 0.001, and ****P < 0.0001. Neut, Neutrophils

PLTs have previously been identified as a threshold switch for NET formation [18, 22, 28, 29]. Then, we examined the potential role of PLTs in NET formation in CVST patients. NPAs (neutrophil-platelet aggregates), defined as CD41^+^/CD66b^+^ cells, were detected in blood samples from heathy controls and CVST patients analyzed by flow cytometry. The results showed that NPAs were obviously increased in samples from CVST patients, especially in samples from the venous sinus thrombus (Fig. 3a). Then, control neutrophils from healthy subjects) were incubated with PRP from CVST patients and healthy individuals. Confocal images revealed significant increases in NETs formation in neutrophils incubated with plasma from CVST patients, especially those from sinus thrombosis sites, compared with those from peripheral venous plasma from healthy subjects (Fig. 3b–f).

PF4 (CXCL4) is stored in PLT granules and has been reported to contribute to thrombosis in previous studies [16, 17]. Recent studies have reported the potential role of PF4 in immune thrombosis in CVST patients who received the COVID-19 vaccine [14, 15]. PF4 from activated PLTs has also been identified as a critical and novel initiator of NET formation [18]. Hence, we investigated the potential role of PF4 in NET formation in CVST patients. The ELISA results showed that plasma PF4 levels showed a significant elevation in plasma samples from CVST patients (Fig. 3g). To further evaluate the function of PF4 in NETosis, control neutrophils were treated with recombinant PF4 protein, and NET formation was evaluated by detecting the concentration of citH3-DNA. The results showed that PF4 promoted NET formation in a dose-dependent manner (Fig. 3h). In inhibition assays, control neutrophils (from healthy subjects) were treated with PRP from healthy controls and CVST patients in the presence of anti-PF4 antibody. Flow cytometry and confocal images results demonstrated that anti-PF4 antibodies could markedly attenuate NET generation induced by PRP from CVST patients (Fig. 3i–o). Our findings validated that PF4 from PLTs was a critical mediator of NET formation in CVST patients.

**Fig. 4:**
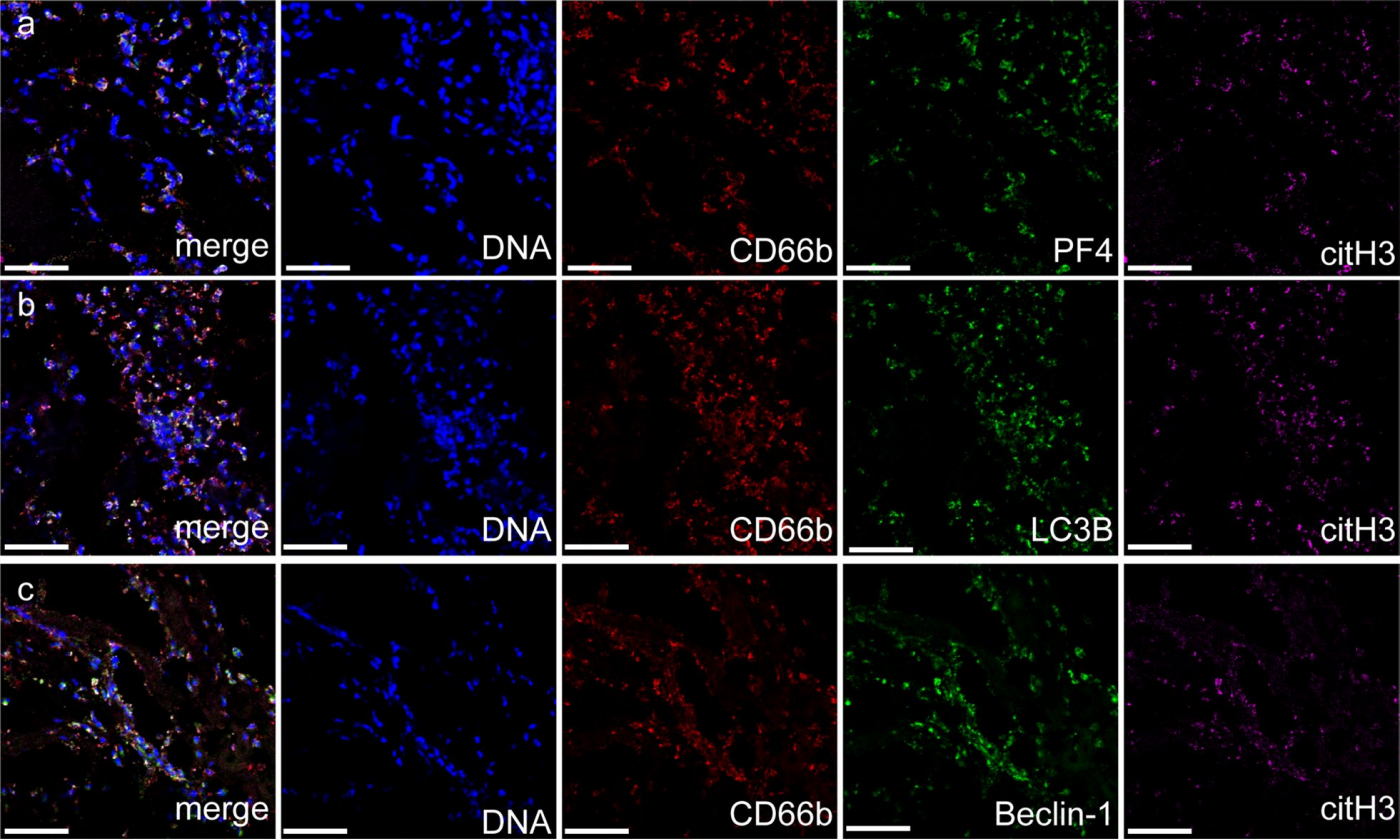
NET markers were colocalized with autophagy associated protein. **a**–**c** CVST thrombi were stained with CD66b (red), citH3 (green), PF4 (pink), Beclin-1 (pink) and LC3B (pink). The inset scale bars are 20 μm in a-c

**Fig. 5:**
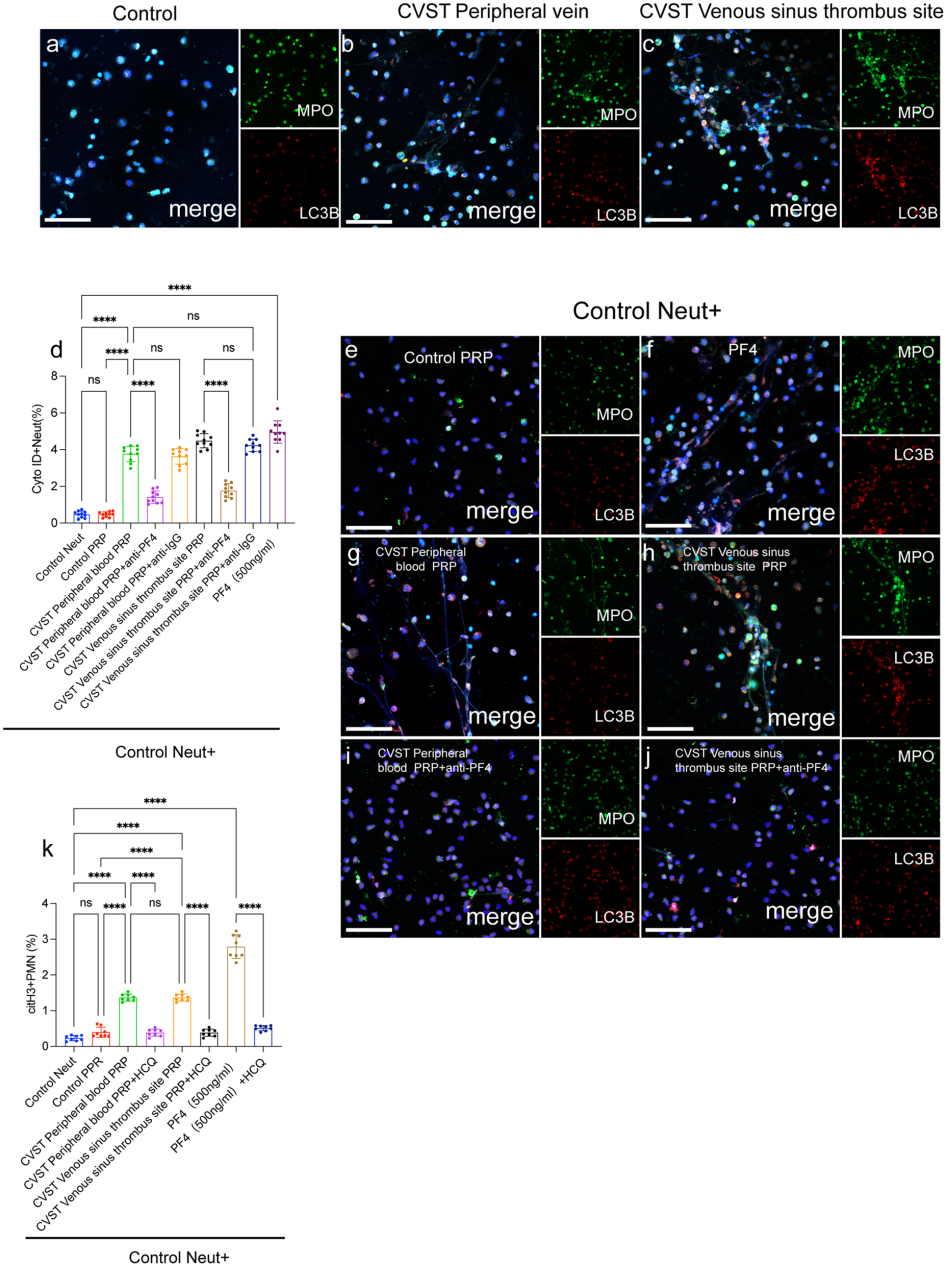
PF4 induce NETs generation through autophagy. **a**–**c** Neutrophils from healthy people and CVST patients were stained with LC3B (red), MPO (green). Control neutrophils were incubated with PRP from healthy subjects and CVST patients in the presence of an anti-PF4 antibody. **d** Neutrophils underwent autophagy and were defined as Cyto ID^+^ cells by flow cytometry. **e**–**j** Neutrophils from each group were co-stained with LC3B (red) and MPO (green) and observed by confocal microscopy. Neutrophils were incubated with PRP from each group and HCQ (autophagy inhibitor). **k** The rate of citH3 + PMN from each group was measured by flow cytometry. Statistics: Brown-Forsythe and Welch’s ANOVA test (D); Kruskal–Wallis test (**k**); The inset scale bars are 20 μm in **a**–**c** and **e**–**j**. ****P < 0.0001

Neutrophil autophagy is a critical mechanism associated with the release of extracellular DNA traps in some cases [29–35]. Therefore, we further investigated the interaction between autophagy and NET formation in CVST. Confocal images showed high expression of PF4, LC3B and Beclin-1 in CVST thrombi, and these markers were colocalized with NET markers (CD66b and citH3) (Fig. 4a–c). This interesting finding provides some hints about the possible role of autophagy in NET formation mediated by PF4. Based on this observation, we measured the expression of autophagy markers in neutrophils from healthy people and CVST patients. Confocal images showed high expression of autophagic proteins in neutrophils from CVST patients, especially in samples from venous thrombus sites (Fig. 5a–c). In addition, we examined the autophagy levels in neutrophils incubated with PRP from the different groups and recombinant PF4 protein. We found that neutrophils incubated with PRP from CVST patients underwent autophagy and were defined as Cyto ID^+^ cells by flow cytometry (Fig. 5d). Confocal images also revealed that PRP from CVST patients activated neutrophils to release NETs with high expression of LC3B (Fig. 5e–j). Moreover, these effects were markedly attenuated in the presence of an anti-PF4 antibody. We further investigated the role of autophagy in NETs generation mediated by PF4. The elevation of NET releasing cells (citH3 + PMN) induced by PF4 could be markedly attenuated in the presence of HCQ (autophagy inhibitor) (Fig. 5k). These results suggested that PF4 induces NET formation through autophagy in CVST.

### NETs enhance procoagulant activity (PCA) in CVST patients

**Fig. 6:**
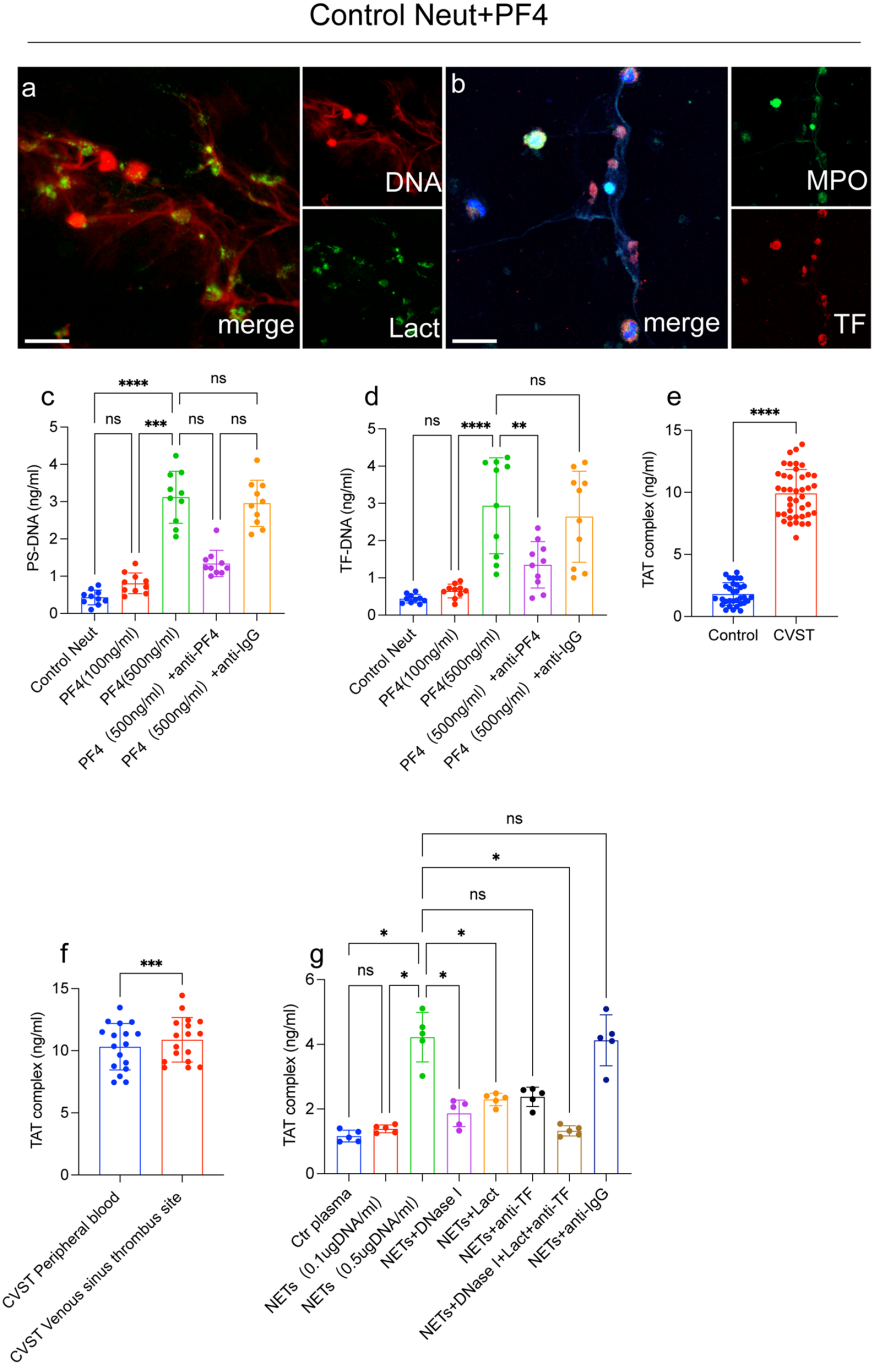
NETs induce PCA in CVST. Control neutrophils were incubated with recombinant PF4 protein. NET structures were decorated with PS **a** and TF (**b**). The supernatant levels of PS-DNA **c** and TF-DNA **d** from neutrophils incubated with different concentrations of PF4 protein. **e** The levels of TAT complexes in plasma from healthy volunteers (n = 32) and patients (n = 37) were measured by ELISA. **f** TAT complexes were measured in peripheral blood plasma (n = 17) and the venous sinus thrombus site from the same patients (n = 17). Control plasma was incubated with isolated NETs (0.5 μg DNA/ml) and treated with lactadherin, anti-TF, and DNase I. **g** TAT complexes were significantly decreased after incubation with DNase I, lactadherin, and an anti-TF antibody. Statistics: Kruskal–Wallis test (**c**, **d**); Unpaired t test with Welch ‘correction (**e**); Wilcoxon t test (**f**). Brown-Forsythe and Welch’s ANOVA test (**g**). The inset scale bar in a and b is 20 μm. *P < 0.05, **P < 0.01, ***P < 0.001 and ****P < 0.0001

The expression of TF was accompanied by autophagy-mediated NET formation in previous studies [29–35]. Interestingly, in our study, when neutrophils were incubated with recombinant PF4 protein, NET structures were decorated with PS and TF (Fig. 6a, b). The ELISA results showed that the expression of PS-DNA (Fig. 6c) and TF-DNA (Fig. 6d) was dependent on PF4 concentrations. These results suggest that NET generation in CVST patients was accompanied by exposure of PS and high expression of TF in DNA traps, suggesting the thrombotic role of NETs in patients with CVST. To investigate the procoagulant role of NETs in CVST patients, the TAT complex (thrombin generation) in plasma samples from each group was measured by ELISA (Fig. 6e, f). The results of ELISA validated that plasma levels of the TAT complex were markedly elevated in samples from patients, especially those from venous sinus thrombus site (Fig. 6e, f). In inhibition assays, DNase I, lactadherin, and anti-TF antibody were used to dissolve DNA traps, PS, and TF, respectively. These inhibitors could markedly diminished thrombin generation, as indicated TAT complex levels (Fig. 6g). Our results demonstrated that NETs could augment the generation of thrombin generation, and these effects could be mitigated by NET inhibitors.

### NETs induce HBMEC thrombogenicity to exacerbate PCA

**Fig. 7:**
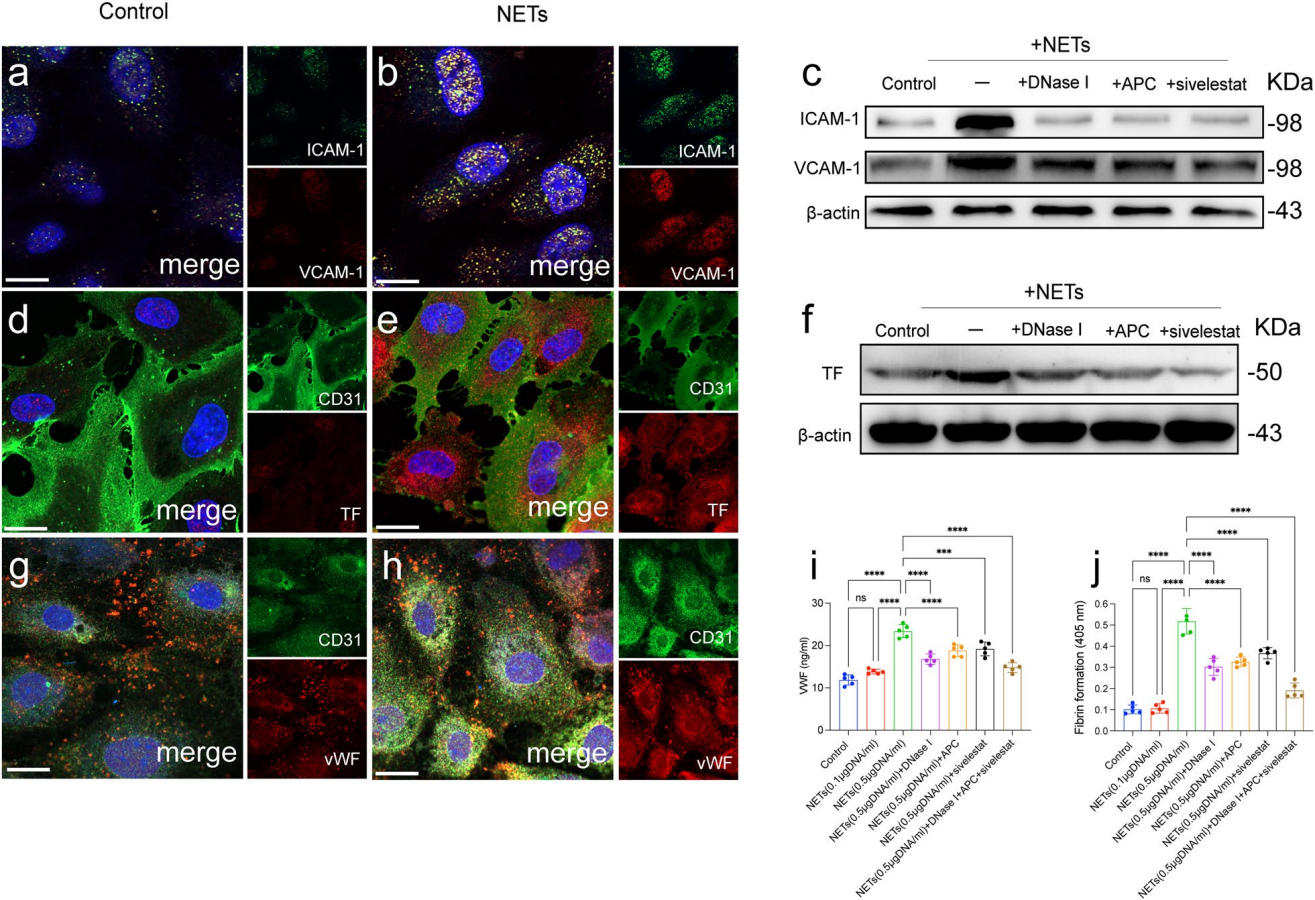
NETs induce thrombogenicity in HBMECs. HBMECs were incubated with isolated NETs in the presence or absence of DNase I, APC and sivelestat. **a**, **b** Representative images showing ICAM-1 (red) and VCAM-1 (green) in treated ECs as observed by confocal microscopy. **c** The expression of ICAM-1 (red) and VCAM-1 (green) was measured by western blotting. **d**, **e** Representative images showing TF (red) and CD31 (green) expression on ECs. **f** The expression of TF on ECs was measured by western blotting. **g**, **h** Representative images showing VWF (red) and CD31 (green) expression on ECs incubated with NETs. **i** The expression of VWF in the supernatant in each group. **(j)** Fibrin formation in ECs incubated with NETs (0.5 μg/mL) in the presence of DNase I, APC and sivelestat. Statistics: Ordinary one-way ANOVA (i, j). The inset scale bars in **a**, **b**, **d**, **e**, **g** and **h** are 20 μm. ***P < 0.001.****P < 0.0001

**Fig. 8:**
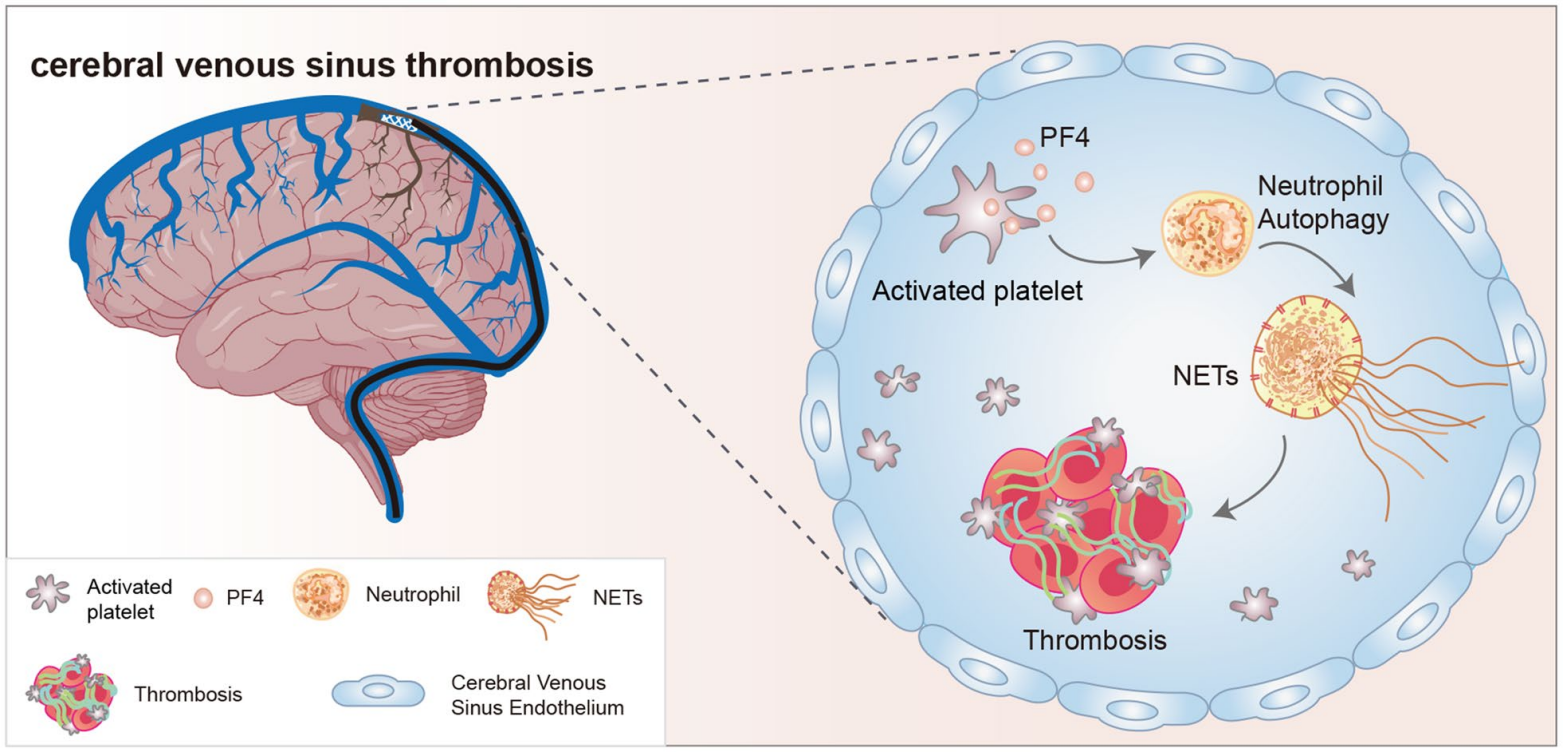
The proposed mechanism of NET formation and its role in PCA in CVST patients

Endothelial dysfunction is a critical process associated with thrombosis [20, 21]. Next, we investigated the role of NETs in HBMEC activation in CVST. HBMECs were treated with isolated NETs. Confocal images and western blotting demonstrated that NETs markedly promote the expression of ICAM-1 and VCAM-1, and this activation state could be attenuated by NETs inhibitors (DNase I, APC, sivelestat) (Fig. 7a–c). Moreover, we investigated the PCA of ECs in the presence of NETs. Confocal images and western blotting showed significantly elevated expression of TF in NET-treated ECs (Fig. 7d, f). In addition, the levels of VWF were significantly elevated in the culture supernatant of ECs incubated with NETs (Fig. 7g–i). PCA was further assessed by evaluating fibrin formation in ECs. We found that increased fibrin formation correlated with NET concentrations. Furthermore, the dissolution of NETs by DNase I, APC, and sivelestat diminished fibrin formation by ECs (Fig. 7j). Figure 8 shows the proposed mechanism of NET formation and its role in PCA in CVST patients.

## Discussion

Four interesting findings were obtained. First, NETs were abundant in thrombi from CVST patients. Second, NETs formation was markedly increased in plasma samples from CVST patients, especially those from the venous sinus thrombus site. Third, PF4 induced NET formation through autophagy in CVST patients. Fourth, NETs activated HBMECs into a procoagulant phenotype to exacerbate thrombosis in CVST patients.

NETs are composed of extracellular chromatins with neutrophil granule proteins such as MPO and NE [9]. In recent two decade, NETs have been identified as an important player in thrombus formation and may be a potential and novel therapeutic target for thrombolysis [11, 12]. In our present study, immunohistochemical staining showed abundant NETs in thrombi from CVST patients. A recent study reported the presence of NETs in thrombi from CVST patients who received the COVID-19 vaccine [14, 15] for the first time. However, the sample size in this study was relatively small (5 patients), and the CVST patients enrolled were only those who received the COVID-19 vaccine, which may have resulted in limiting the conclusions. In our study, we obtained 20 thrombi from CVST patients who underwent thrombectomy. In ex vivo experiments, the combination of DNase I and tPA markedly promoted lysis compared to tPA or DNase I alone, suggesting that NETs may be potential therapeutic targets for CVST. In addition, we further investigated the plasma NET levels in CVST patients. The plasma levels of NET markers suggested a marked elevation in CVST patients, especially in samples from the venous sinus thrombus site, suggesting the potential prothrombotic role of NETs in CVST. Flow cytometry also showed a similar trend as ELISA result. Our present findings not only demonstrated the increased generation of NETs in CVST formation, but also the differences of NETs distribution in peripheral blood and venous sinus thrombus site. Further studies should explore the mechanism of this interesting observation.

PLTs have been considered critical mediators of NET formation in some diseases [22, 28, 29]. For example, activated PLTs release HMGB1 and PF4 to induce the generation of NETs in thrombotic diseases such as stroke [36], venous thromboembolism (VTE) [28], and sepsis [29]. Our results showed increased levels of NPAs in samples from the venous sinus thrombus site. Moreover, a PLT-neutrophil coincubation assay revealed that PRP from CVST patients was a potent activator of NET generation. Furthermore, we investigated the potential mechanism of PLT activation in NETs formation in CVST. Recent studies have reported the potential role of PF4 in immune thrombosis in patients who received the COVID-19 vaccine [14, 15]. PF4 from activated PLTs has also been shown to induce NET formation in some diseases [18]. Therefore, we investigated the presence and potential role of PF4 in CVST patients. The ELISA results showed that the levels of PF4 were markedly increased in plasma samples from CVST patients, especially those from the venous sinus thrombus site. Our results showed that PF4 promoted NET generation in a dose-dependent manner. It was previously shown that PF4 delayed apoptosis. Moreover, after neutrophils were activated following incubation with a recombinant PF4 protein, they expelled extracellular traps decorated with PS and TF on DNA structures.

Autophagy in activated neutrophils was a critical mechanism for the release of extracellular DNA traps in previous reports [29]. Interestingly, in our study, we observed the presence of abundant NETs that colocalized with PF4 and autophagic proteins. Therefore, we investigated whether PF4-induced NET formation was mediated by autophagy. Flow cytometry and confocal images showed that PLTs from the venous sinus thrombus site activated neutrophils to release NETs, and these effects were markedly attenuated in the presence of anti-PF4 antibodies or HCQ (autophagy inhibitor), demonstrating that PF4 enhances the generation of NETs through autophagy.

Neutrophils have previously been shown to play an important role in thrombus formation, and NETs promote both venous and arterial thrombosis [11–13]. In our study, abundant NETs were observed in thrombi and in plasma samples from CVST patients. In addition, the concentration of the TAT complex, a marker of thrombin generation, was markedly elevated in samples from CVST patients. The TAT complex was also positively correlated with NET markers in samples from CVST patients. In vitro studies showed that the generation of thrombin was markedly elevated in plasma incubated with isolated NETs. TF is an important initiator of the extrinsic coagulation cascade and is highly expressed during NET formation. Our study also showed that PF4 promoted neutrophils to generate extracellular traps decorated with TF and PS. In inhibition assays, lactadherin and anti-TF antibodies inhibited PS and TF. Furthermore, these inhibitors could reverse the PCA of NETs.

ECs play an important role in vascular homeostasis and the occurrence and development of thrombosis [37]. In the present study, we established a NET-HBMEC coculture system to investigate the interaction between NETs and EC activation. The present study showed that NETs from CVST patients enhanced ICAM-1 and VCAM-1 expression in ECs. Previously, we showed that histones could destroy the endothelial barrier. Our results demonstrated that NE participates in the degradation of ECs. NETs caused the hyperexpression of TF in ECs in our findings. The ELISA results showed that NETs not only disrupted the endothelial barrier but also induced the release of VWF by ECs. VWF has been shown to play a central role in thrombotic diseases such as stroke [38]. According to our results and previous studies, the crosstalk between NETs and VWF may be critical in thrombosis in CVST patients. Future studies should further investigate the potential mechanism between VWF and NET formation in thrombosis in CVST patients.

However, our present study has certain limitations. First, our clinical data and samples were based on a relatively small sample size due to the low occurrence of CVST. Our future studies with larger sample sizes from multiple centers will refine our present findings. Second, our study obtained samples from clinical patients in two hospitals and preliminarily conformed presence and the procoagulant role of NETs in CVST patients. Thus, in vivo experiments with animal models should be considered in the future to gain insight to specific mechanism of the interaction between CVST and NETs.

In conclusion, we have identified that NETs are involved in hypercoagulation and thrombus composition in CVST patients. We also demonstrated that PF4 from CVST patients induces NET formation through autophagy. Furthermore, the crosstalk between NETs and ECs exacerbated thrombogenicity in CVST patients. Our findings may provide new therapeutic targets to both prevent and combat CVST complications.

## Supplementary Information

**Additional file 1.**

## Data Availability

The data required to reproduce these findings cannot be shared at this time due to technical or time limitations. Data are available from the corresponding author upon reasonable request after article published.

## Abbreviations

NETs: Neutrophil extracellular traps
CVST: Cerebral venous sinus thrombosis
PCA: Procoagulant activity
citH3: Citrullinated histone H3
NE: Neutrophil elastase
PLT: Platelet
PF4: Platelet factor 4
ECs: Endothelial cells
VWF: Von Willebrand factor
APC: Activated protein C
BBB: Blood–brain barrier
PS: Phosphatidylserine
TF: Tissue factor
PFA: Paraformaldehyde
PRP: Platelet-rich plasma
EDTA: Ethylenediaminetetraacetic acid -anticoagulated
MPO: Myeloperoxidase
TAT: Thrombin-antithrombin complex
ELISA: Enzyme-linked immunosorbent assay
