# Supplementary material for: Neutrophil extracellular traps promote thrombogenicity in cerebral venous sinus thrombosis
Source: Cell Biosci. 2022 Jul 22;12:114. doi: 10.1186/s13578-022-00845-z (PMC9306243; doi:10.1186/s13578-022-00845-z)
Supplement: Supplementary file 1 — Additional file 1. [file 13578_2022_845_MOESM1_ESM.docx]

**Supplementary methods**

**NETs isolation**

Briefly, isolated neutrophils (5 x 10^6^ neutropils/ml) from healthy subjects or patients and were incubated with 500 nM PMA for 4 h. In order to lift off all adherent NETs from the bottom, we removal of the supernatant and wash the bottom of each dish by pipetting 15 ml of cold PBS on the bottom of the dish and collected solution, centrifuge for 10 min at 450 x g at 4 °C. Then, we divided supernatant into 1.5 ml micro-centrifuge tubes and centrifuge for 10 min at 18,000 x g at 4 °C. Measure DNA concentration in isolated NETs (μgDNA/ml) using spectrophotometry and we stored isolated NETs at -80°C for further experiment.

**Supplement Figure 1**

Flow cytometry analysis of NETing neutrophils(FITC+/APC+ group) and platelet-neutrophils(Percp-Cy5.5+/APC-Cy7+group) aggregates from whole blood.

**
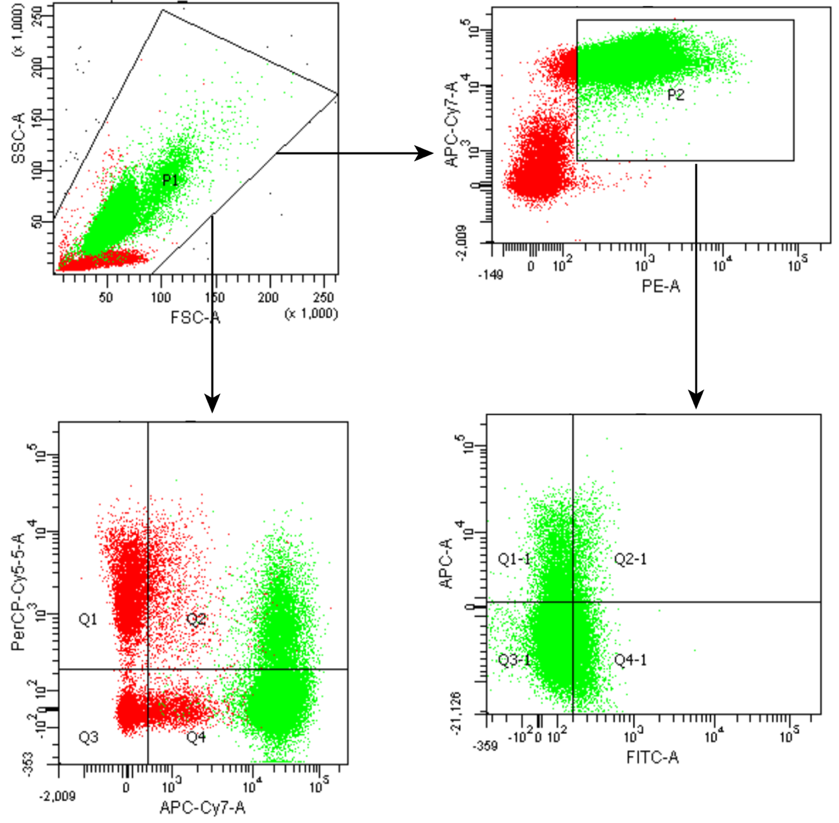
**

**Supplement Figure 2**

CVST thrombi were from patients underwent thrombectomy and in different venous sinus(Superior sagittal sinus, n=10;Transverse sinus, n=3; Sigmoid sinus, n=4). Neutrophil numbers(a) and citH3-positive signal in CVST thrombi (n=17) were quantified. Statistics: Ordinary one-way ANOVA.

**
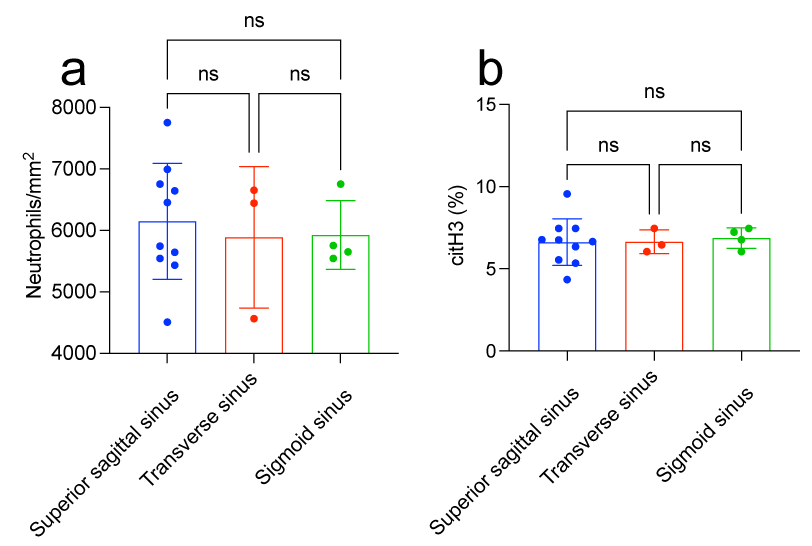
**
